# Prognostic value of 24-hour cultivation of peritoneal fluid to distinguish complicated from uncomplicated acute appendicitis: a prospective cohort study

**DOI:** 10.1007/s00423-024-03428-3

**Published:** 2024-08-08

**Authors:** Anders Mark-Christensen, Ditte Bro Sørensen, Niels Qvist, Ulrik Stenz Justesen, Sören Möller, Mark Bremholm Ellebæk

**Affiliations:** 1 Department of Surgery, Odense University Hospital, Odense, Denmark; 2 Department of Clinical Microbiology, Odense University Hospital, Odense, Denmark; 3 Open Patient data Explorative Network, Odense University Hospital, Odense, Denmark; 4 Research Unit OPEN, Department of Clinical Research, University of Southern Denmark, Odense, Denmark

**Keywords:** Acute appendicitis, Cultivation, Antibiotics, Prospective cohort study

## Abstract

**Background:**

The distinction between complicated and uncomplicated acute appendicitis (AA) is important as it guides postoperative antibiotic treatment. A diagnosis based on intraoperative findings is imprecise and standard cultivation of peritoneal fluid is generally time-consuming with little clinical benefit. The aim of this study was to examine if cultivation of peritoneal fluid in acute appendicitis could reliably detect bacteria within 24 h.

**Methods:**

Patients older than 18 years undergoing laparoscopic appendectomy were prospectively enrolled at two surgical departments after informed consent was obtained. Periappendicular fluid was collected prior to appendectomy and sent for cultivation. Sensitivity, specificity and positive and negative predictive values were calculated with 95% confidence intervals (CIs) using 72-hour cultivation results as the gold standard. Patients with complicated AA as determined by the surgeon, received a three-day course of oral antibiotics. Postoperative infectious complications within 30 days after surgery were registered.

**Results:**

From July 2020 to January 2021, 101 patients were included. The intraoperative diagnosis was complicated AA in 34 cases. Of these patients, six (17.6%) had bacteria cultured within 24 h after surgery, leading to a sensitivity of 60% and a specificity of 100%. The positive and negative predictive values were 1.00 and 0.96, respectively. Seven patients developed a postoperative infection (five superficial wound infections and two intra-abdominal abscess). In all cases with a positive cultivation result, the intraoperative diagnosis was complicated appendicitis and a postoperative course of antibiotics prescribed.

**Conclusion:**

Twenty-four-hour cultivation of the peritoneal fluid in acute appendicitis is a valid indicator for peritoneal bacterial contamination. Randomized studies are necessary to determine if this approach is suitable for targeting postoperative antibiotic treatment as a means to prevent overtreatment without increasing the risk of infectious complications.

## Introduction

The life-time risk of developing acute appendicitis (AA) is 7–9% [1] and is among the most common emergency conditions encountered in general surgery. Based on intraoperative findings, AA is typically categorized as uncomplicated or complicated, although there is no universally agreed upon definition of complicated AA. Gomes et al [2] have suggested a 5-graded classification based upon the findings at laparoscopy with grade 1–2 (normal looking appendix, hyperemia and edema of the appendix and fibrinous exudate) as defining uncomplicated AA and grade 3–5 with any gangrene of the appendix, abscess and/or peritonitis as complicated AA. Another 5-graded system including perforation has been suggested by others [3]. The distinction between uncomplicated and complicated AA is important, because the postoperative regimen following appendectomy for complicated AA usually dictates a course of intravenous antibiotics to prevent infectious complications. A previous study found that in 42% of cases, where the surgeon deemed the condition as complicated during surgery based on intraoperative findings, no bacteria could be cultivated from the peritoneal fluid [4]. This may lead to both unnecessary use of antibiotics, which increases the risk of developing multi-resistant bacteria, and prolonged hospitalizations owing to in-hospital treatment with intravenous antibiotics. Most guidelines recommend a single 24-hour dosage of prophylactic antibiotics perioperatively when performing an appendectomy [5], which leaves a diagnostic window to investigate the peritoneal fluid collected at laparoscopy for bacterial contamination as an indication for continued antibiotic treatment postoperatively.

A prior observational study has found that oral antibiotic treatment is non-inferior to intravenous treatment [6], and, contrary to intravenous treatment, oral treatment can be administered on an out-patient basis, potentially reducing the costs associated with treatment of complicated appendicitis.

The primary aim of this study was to determine if cultivation of the peritoneal fluid in cases of acute appendicitis was feasible and could yield reliable results within 24 h after appendectomy. The secondary aims were to compare results of the peritoneal cultivation in relation to the surgeon’s diagnosis of uncomplicated or complicated AA and to evaluate the rate of postoperative infections within 30 days postoperatively in a setting where patients with complicated AA were treated with oral antibiotics for three days after surgery.

## Methods

We used a prospective cohort design, where patients were included in the emergency departments at two hospitals (Svendborg and Odense University Hospital) on the island of Funen in Denmark. Patients were recruited from a background population of approximately 500,000 individuals in the period from July 2020 to January 2021.

Patients older than 18 years undergoing a diagnostic laparoscopy on suspicion of AA were screened for eligibility and received verbal and written information regarding the study prior to surgery. Patients were excluded if they had received antibiotics prior to surgery, if they were pregnant or if they were cognitively impaired, whether it was due to chronic disease (e.g., dementia) or acute illness (e.g., organic delirium). Further, if patients underwent a surgical procedure other than laparoscopic appendectomy (e.g., open appendectomy, excision of Meckel’s diverticulum, etc.), they were excluded post hoc.

Based on an assumption that approximately one third of patients undergoing laparoscopic appendectomy will be diagnosed with a complicated AA, we considered inclusion of 100 patients as sufficient to calculate validity of peritoneal cultivation. No formal sample size calculations were performed. The study was approved by the National Committee of Health Research Ethics (reference number S-20,190,186) and the Danish Data Protection Agency (reference number 20/1691). The study protocol was registered at clinicaltrials.gov (NCT04713527) prior to inclusion of the first patient.

### Study intervention

#### Laparoscopic appendectomy, peritoneal lavage and postoperative antibiotic treatment

Diagnostic laparoscopy was typically performed by either residents or surgical fellows using three ports; one for optics, one for a laparoscopic grasper and one for energy device and extraction bag. Placement of ports was performed at the operating surgeon’s discretion.

Once AA was diagnosed, any periappendiceal pus or fluid was collected by suction device and sent for cultivation. If there was no significant fluid to collect, right iliac fossa lavage was performed with 10 ml isotonic saline and a sample extracted for cultivation. After peritoneal fluid aspiration, patients received a single dose of antibiotics, typically 1,5 g of metronidazole and 4 g/500 mg of piperacillin-tazobactam as per local guidelines. In some instances, due to local practice or surgeon preference, patients received 3 g of cefuroxime instead of piperacillin-tazobactam.

Appendectomy was then commenced using a combination of electrocautery and dissection devices. The appendix was ultimately divided using either hemostatic clips (Hem-o-lok^®^) or an endoscopic stapling device and extracted in an extraction bag.

When the operating surgeon suspected complicated AA based on intraoperative findings (perforation, local or diffuse peritonitis, gangrene and/or abscess), a 3-day postoperative course of oral antibiotics consisting of 500 mg amoxicillin-clavulanic acid and 500 mg metronidazole three times a day was prescribed. In cases of allergy to penicillin, patients would receive 500 mg ciprofloxacin twice a day instead of amoxicillin-clavulanic acid.

### Primary outcome

#### Validity of 24-hour cultivation of peritoneal fluid

Pus or peritoneal lavage fluid was sent for culture to the local department of clinical microbiology immediately after surgery. Samples were cultured and evaluated for bacterial growth after 24 h and three days. We defined the result at three days as the gold standard.

### Secondary outcomes

#### Concordance between intraoperative classification of acute appendicitis and cultivation results

Using the final (72-hour) cultivation result as the gold standard for the distinction between uncomplicated and complicated AA, we also calculated the sensitivity, specificity and positive and negative predictive values of the intraoperative distinction between uncomplicated and complicated appendicitis made by the operating surgeon.

### Postoperative infectious complications within 30 days

Information on infectious complications occurring within 30 days after surgery was collected by chart review and direct contact to patients by telephone interview by a study investigator (DS). Infectious complications included superficial wound infections managed expectantly or opened at bedside and intraabdominal abscesses, whether these were managed conservatively with antibiotics or by image-guided drainage or surgery.

### Covariates

Data on the following baseline covariates were collected from hospital charts: *American Society of Anesthesiologists* (ASA) grade, *body mass index* (BMI; kilograms/height (m)^2^), smoking status (yes/no) and alcohol consumption (below or above 7 (women)/14 (men) units of alcohol per week).

### Statistical analysis

Categorical data are presented as numbers (percent) while continuous data are presented with means (standard deviations) or medians (interquartile range), depending on distribution. Differences in baseline covariates and outcomes between patients with uncomplicated and complicated AA were tested using either Student’s t-test (for continuous data) or x^2^ (for categorical data). A p-value < 0.05 was considered statistically significant. We calculated sensitivity of the 24-hour observation as the proportion of true positive cultivations divided by the sum of true positive and false negative cultivations (where the 3-day cultivation revealed bacteria not found within 24 h). Specificity was calculated as the proportion of true negative cultivations divided by the sum of true negative and false positive cultivations. The negative predictive value was calculated as the proportion of true negatives divided by the sum of true negatives and false negatives, while the positive predictive value was calculated as the proportion of true positives divided by the sum of true positive and false positives.

## Results

**Fig. 1:**
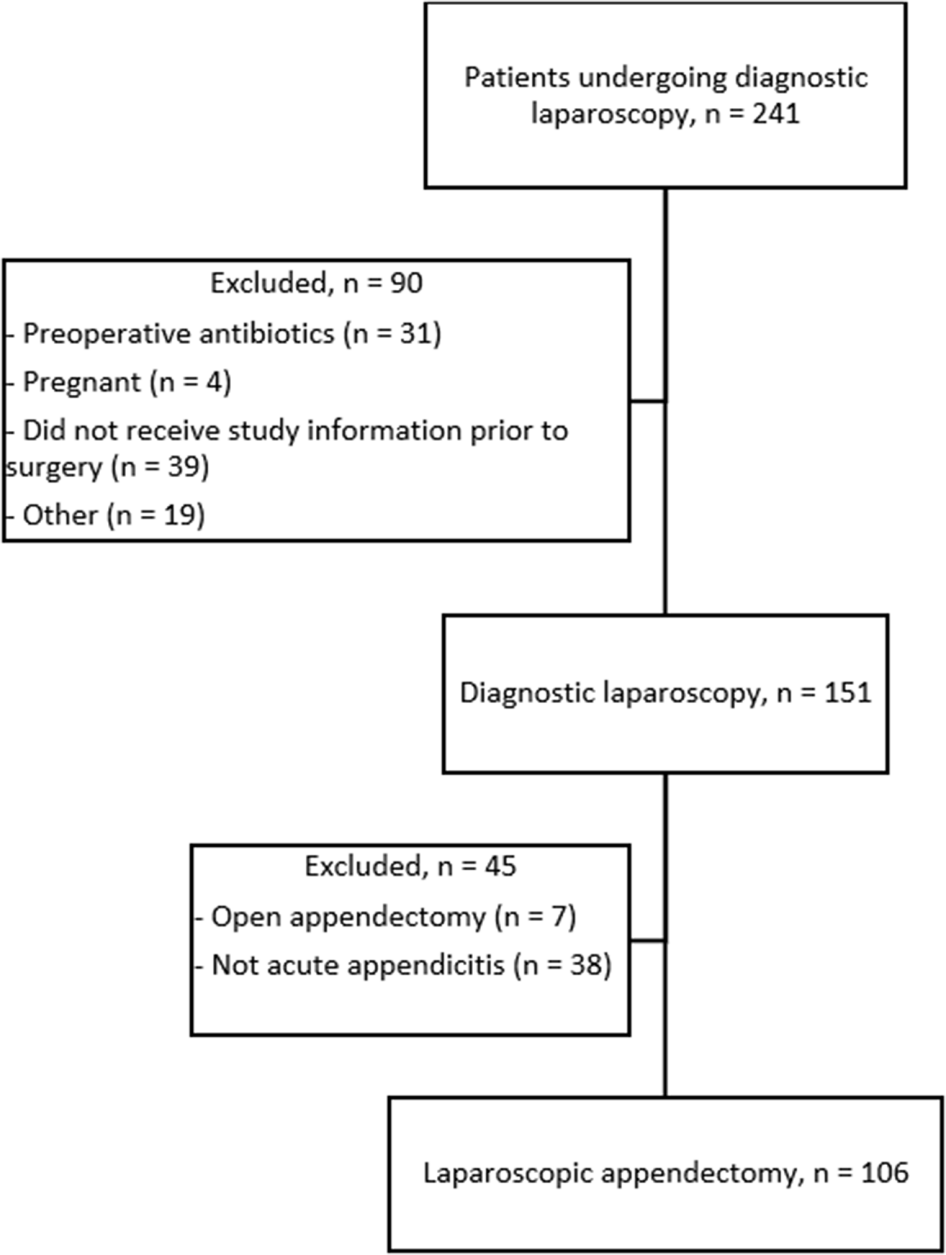
Study flowchart

During the study period, 241 patients 18 + years underwent diagnostic laparoscopy on suspicion of AA (see Flow chart, Fig. 1). Of these, 31 had received antibiotics prior to surgery, four were pregnant, 16 fulfilled at least one exclusion criterium and 39 cases fulfilled inclusion criteria, but were not included due to logistical issues and were thus considered missed opportunities for inclusion prior to surgery. Of the 151 patients included prior to surgery, seven underwent open appendectomy and 38 patients did not have AA (these cases included diagnostic laparoscopies with normal findings, gynecological conditions, etc.). This left 106 patients with AA undergoing laparoscopic appendectomy with intraoperative, pre-appendectomy fluid or pus collection.

In five cases, the observation of cultivation samples for bacterial growth began more than 36 h after appendectomy. These samples were excluded from analysis, because the timing of bacterial growth could not be determined. In one of these five cases, the surgeon defined the appendicitis as uncomplicated, although bacteria were detected on final cultivation results.

Baseline data on the 101 patients whose peritoneal samples provided the basis for calculations of diagnostic validity of 24-hour cultivations are listed in Table 1. Overall, patients with complicated AA were older, had more comorbidities than patients with uncomplicated AA and there was a higher proportion of males in this group.

**Table 1:** Baseline covariates for patients with acute appendicitis, stratified in complicated and uncomplicated AA based on intraoperative findings

Baseline covariate	Complicated AA	Uncomplicated AA	Total
**Number**	34	67	101
**Age at surgery**,** median (IQR)**	53.0 (38.9–74.0)	31.8 (23.8–68.2)	38.7 (25.9–75.3)
**Female sex**,** n (%)**	14 (41)	35 (52)	49 (48.5)
**BMI**,** median (IQR)**	26.3 (24.3–32.5)	25.1 (22.7–35.4)	26.0 (23.0-35.4)
**ASA grade**,** n (%)**			
- **I**	13 (38)	37 (55)	50 (50)
- **II**	19 (56)	29 (43)	48 (48)
- **III**	2 (6)	1 (1.5)	3 (3)
**Smoking: n (%)**			
- **Current**	8 (24)	15 (22)	23 (23)
- **Former**	7 (21)	12 (18)	19 (19)
- **Never**	18 (53)	40 (60)	58 (58)
- **Unknown**	1 (3)	0	1 (1)
**Current alcohol use**,** n (%)**	2 (6)	7 (10)	9 (9)

Abbreviations: AA; acute appendicitis, IQR; interquartile range, BMI; body mass index, ASA; American Society of Anesthesiologists

From the 101 patients included, 34 were considered to have complicated AA based on intraoperative findings by the surgeon, and six of these had a positive 24-hour cultivation. The bacteria cultivated within 24 h from the six positive samples were *Staphylococcus warneri*,* Streptococcus anginosus*,* Bacteroides ovatus*,* Bacteroides thetaiotaomicron and Pseudomonas aeruginosa.*

In four cases, 24-hour cultivations were negative while final cultivations were positive. The bacteria found in these instances were *Streptococcus anginosus*,* Bacteroides caccae*,* Bacteroides vulgatus*,* Bacteroides salyersiae and Pseudomonas aeruginosa*. Thus, in only 10 of 34 cases of complicated AA, the cultivation revealed bacterial growth. There was one case of a positive final cultivation, where the intraoperative diagnosis was uncomplicated appendicitis.

Table 2 provides the numbers relating to the positive and negative findings on 24-hour and 3-day cultivations. From these numbers, the sensitivity of 24-hour cultivation was 60% and specificity 100%. The positive predictive value was 1 and the negative predictive value was 0.96.

**Table 2:** Diagnostic validity of 24-hour peritoneal cultivation compared to 3-day cultivation results

	3-day cultivation: Positive	3-day cultivation: Negative	Total
**24-hour cultivation: Positive**	6	0	6
**24-hour cultivation: Negative**	4	91	95
**Total**	10	91	101

The numbers relating to the validity of the intraoperative diagnosis of complicated AA are listed in Table 3; sensitivity was 93,3%, specificity 73,6%, positive predictive value 0.37 and negative predictive value 0.99. Because these calculations only relied on final cultivation results and the intraoperative distinction between complicated and uncomplicated AA, the five cases with no “early” cultivation results were included in this analysis.

**Table 3:** Diagnostic validity of surgeon’s intraoperative diagnosis and final (3-day) cultivation results

	Cultivation: Complicated	Cultivation: Uncomplicated	Total
**Surgeon: Complicated**	14	24	38
**Surgeon: Uncomplicated**	1	67	68
**Total**	15	91	106

Of the 34 patients with complicated AA as determined by the operating surgeon, 30 received a three-day postoperative course of oral antibiotics and four received intravenous antibiotics.

**Fig. 2:**
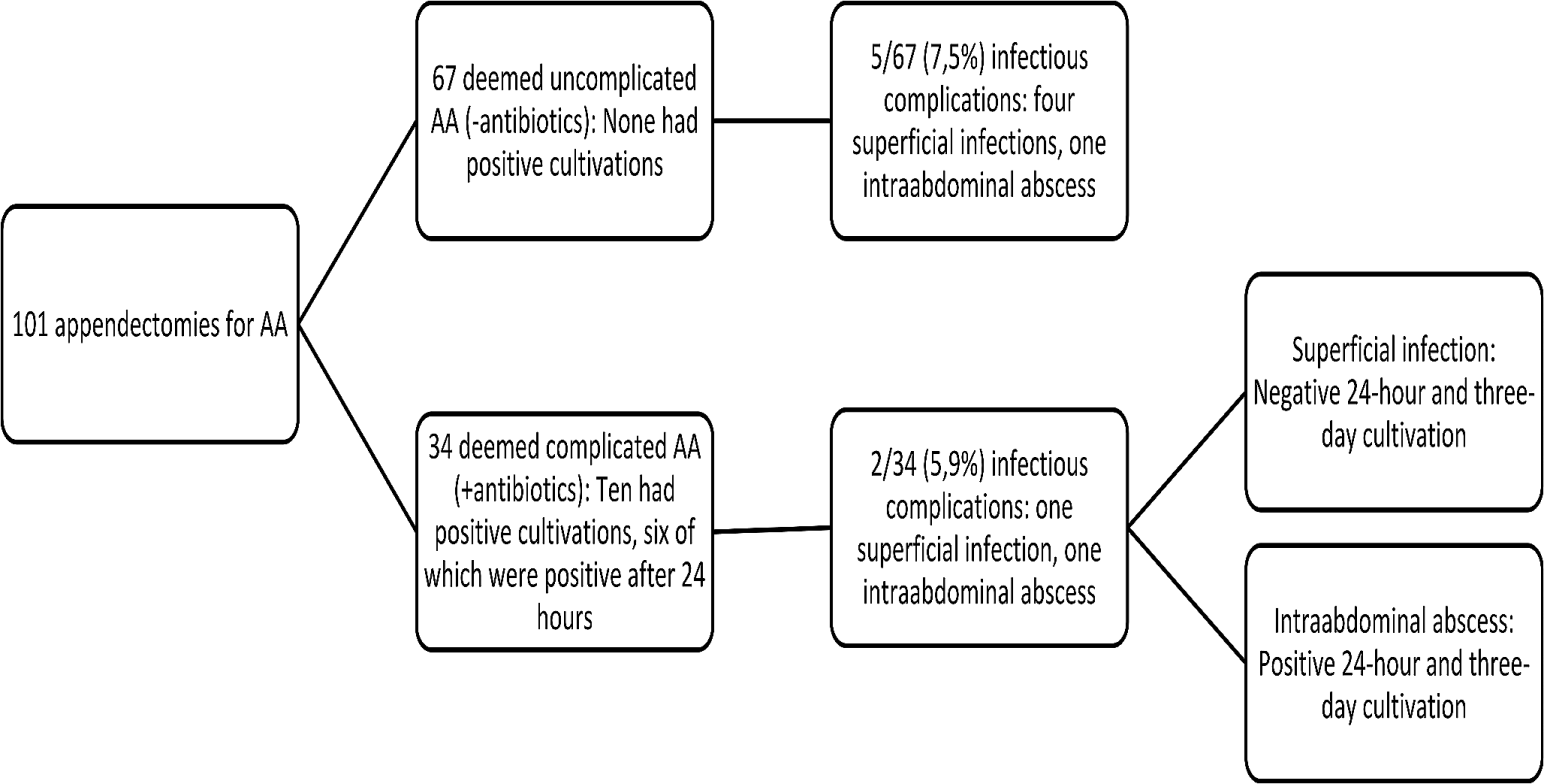
Rates of infectious complications according to intraoperative diagnosis and 24-hour cultivation results for patients with acute appendicitis and a valid 24-hour cultivation. Abbreviations: AA, acute appendicitis

Figure 2 illustrates rates of postoperative infectious complications based on 24-hour cultivation results and intraoperative findings. A total of seven infectious complications were reported within 30 days after laparoscopic appendectomy, five of which occurred after an operation for uncomplicated AA as judged both by the surgeon and the absence of bacteria on cultivation. Of the seven complications, five were superficial wound infections, while two were intraabdominal abscesses.

Of the four patients with documented iatrogenic perforation of the appendix during appendectomy, three developed infectious complications (one superficial infection and two intraabdominal abscesses). All these patients received postoperative antibiotics, but there were no positive cultivations because samples were drawn prior to the perforations. In two of the four cases, the appendix was described gangrenous and in the other two, it was described uncomplicated.

## Discussion

An intraoperative distinction between uncomplicated and complicated AA is difficult and grossly overestimates the true prevalence of complicated AA [4], leading to unnecessary antibiotic use and prolonged hospitalization. With this study, we found that 24-hour cultivation of periappendiceal fluid collected immediately before laparoscopic appendectomy could reliably determine the presence of bacteria as evident from a high negative predictive value. This observation has important clinical implications, because an early distinction between uncomplicated and complicated AA potentially can reduce the need for intravenous antibiotic treatment and subsequently the length of hospitalization.

Of the 34 cases of complicated AA diagnosed by the operating surgeon, 24-hour cultivations were positive in six cases and final 3-day cultivations positive in ten, reproducing the previous findings of surgeons overestimating the true prevalence of complicated AA. A previous study based on 144 patients with complicated AA found bacteria in 74% of cases after four days of cultivation when complicated AA was diagnosed during surgery, which is a significantly higher proportion than in our study [7]. This may be a result of different definitions of complicated AA, a higher prevalence of perforated appendicitis and differences in peritoneal sampling methods.

Of the four cases in our study where 24-hour cultivations were negative and final cultivation positive, the intraoperative diagnosis was complicated AA, and so, patients received postoperative antibiotics.

These findings suggest that the adjunctive use of periappendiceal fluid sampling during laparoscopic appendectomy for AA may lead to a more restrictive use of antibiotics, where antibiotics are reserved for the cases where both the intraoperative, macroscopic findings and cultivation results support a diagnosis of complicated AA. However, we did not determine the spontaneous rate of infectious complications for patients with an intraoperative diagnosis of complicated AA and a negative 24-hour cultivation result as they were all treated with antibiotics. The low rate of infectious complications in this group (one in 28), which is even lower than the rate of complications in the group with both a negative cultivation and intraoperative diagnosis of uncomplicated AA, does however suggest a benign course.

Besides the prospect of restricting antibiotic use, an added benefit of peritoneal cultivations is that antibiotic treatment can be targeted the specific microorganisms, although the majority of bacteria found on cultivations were sensitive to the standard empirical postoperative antibiotic treatment.

Interestingly, iatrogenic appendix perforations are not widely considered an indication for postoperative antibiotic prophylaxis. We reported four cases of iatrogenic perforations, where three developed infectious complications, including two intraabdominal abscesses, despite receiving postoperative antibiotics. These findings indicate that iatrogenic perforations could be considered complicated AA on the same line as gangrenous or perforated AAs and that periappendiceal fluid sampling should be performed after appendectomy instead of before.

In addition to the inherent limitations of a non-randomized trial as discussed above, our study was limited by a relatively low number of patients and consequently few cases of complicated AA, which impairs the robustness of our estimates. Nonetheless, our findings are likely generalizable to similar western European tax-funded health care systems as relatively few patients were excluded for reasons that could potentially bias the estimates and patients were recruited from an unselected background population.

Most guidelines recommend the use of intravenous antibiotics for three days after surgery for complicated AA [5]. In our study, all but four patients who were intraoperatively considered to have a complicated AA received oral antibiotics. Although we made no head-to-head comparison of postoperative infectious complications with patients who received intravenous antibiotics, the rate of postoperative infections in our study following laparoscopic appendectomy for complicated appendicitis appears comparable to what has been reported in other studies [8, 9]. In the absence of adequately powered randomized controlled trials, a course of oral antibiotics for patients with gastrointestinal function seems appropriate.

Based on our findings, it can be argued that the slightest suspicion of complicated appendicitis or iatrogenic perforation should merit fluid collection and cultivation, where further treatment could be guided by the results of the 24-hour cultivation, if intraoperative findings also suggest complicated AA. In this regard, it is relevant to recapitulate the fact that the diagnostic validity of 24-hour cultivation (as any other diagnostic test) depends on the prevalence of the examined disease. A higher true prevalence of complicated AA among a selected group of patients with intraoperatively diagnosed complicated AA – as opposed to patients with both uncomplicated and complicated AA – will ultimately lead to a lower negative predictive value of 24-hour cultivations. A randomized study of periappendiceal fluid cultivation could help answer the question of whether postoperative antibiotic treatment for patients with intraoperatively diagnosed complicated AA should be reserved to those with proven intraperitoneal bacterial colonization in order to minimize superfluous antibiotic use and reducing admission lengths without increasing the risk of postoperative infections.

In conclusion, we found that 24-hour cultivation of periappendiceal fluid collected prior to appendectomy for AA yielded reliable results compared with three-day cultivations in a population where complicated AA is relatively infrequent. The adjunctive use of 24-hour cultivation could enable a more restricted and targeted use of postoperative antibiotics. In cases where antibiotic treatment is indicated after surgery, oral administration seems safe and efficient.

## Data Availability

No datasets were generated or analysed during the current study.
